# The performance of free-breathing multiparametric SAturation-recovery single-SHot acquisition T1 and T2 mapping in cardiac allograft rejection

**DOI:** 10.1007/s10554-025-03582-9

**Published:** 2025-12-12

**Authors:** Nikolaos Miaris, Husein Rajabali, Nicholas M Quaife, Fernando Riesgo Gil, Owais Dar, Andrew Morley-Smith, Jan Lukas Robertus, Muhammad Usman, Antonis Pantazis, Rajasi Banerjee, Barbara Segulin, Thomas Luescher, Chiara Bucciarelli-Ducci, Kelvin Chow, Peter Kellman, Joyce Wong

**Affiliations:** 1 Royal Brompton and Harefield Hospitals, Guy’s and St Thomas’ NHS Foundation Trust, London, UK; 2 School of Cardiovascular Medicine and Sciences, King’s College London, London, UK; 3 National Heart and Lung Institute, Imperial College London, London, UK; 4 Cardiovascular MR R&D, Siemens Healthcare Ltd., Calgary, AB Canada; 5 National Heart, Lung, and Blood Institute, National Institutes of Health, Bethesda, MD USA

**Keywords:** Cardiovascular magnetic resonance, Parametric mapping, mSASHA, Heart transplant, Cardiac allograft rejection

## Abstract

Cardiovascular magnetic resonance (CMR) has been studied as an alternative to endomyocardial biopsy (EMB) in orthotopic heart transplant recipients (OHTRs), particularly through breath-hold myocardial T1 and T2 mapping sequences. We aimed to assess the diagnostic performance of the novel free-breathing multiparametric saturation-recovery single-shot acquisition (mSASHA) mapping technique to identify acute cardiac allograft rejection (ACAR) non-invasively against EMB. This retrospective study included *n* = 21 consecutive OHTRs with suspected ACAR who underwent both a 1.5T CMR scan with mSASHA mapping and an EMB within 5.4 (1.2–14.8) days, and *n* = 20 healthy controls who underwent CMR with mSASHA mapping. Conventional breath-holding T2-prepared balanced steady-state free precession (T2p-bSSFP) T2 mapping was also acquired. CAR was EMB defined as histological rejection (HR) (acute cellular rejection of ≥ 1 R or acute antibody-mediated rejection of ≥ 1). Overall, *n* = 21 OHTRs (9 females) with a mean age of 43.2 ± 16.2 years and a median time of 5.4 (1.2–14.8) years since transplantation were included in the study, alongside *n* = 20 healthy controls. HR was present in *n* = 9 patients. The HR group showed significantly higher septal mSASHA T2 values compared to the non-HR (*n* = 12) group (53 ± 6 ms vs. 47 ± 4 ms, *p* = 0.014). Receiver operator characteristics analysis showed an area under the curve of 0.79 (95% confidence interval 0.59–0.98, *p* = 0.028) and an optimal cut-off value of 50 ms (sensitivity of 67%, specificity of 75%) for identifying patients with HR using septal mSASHA T2 values. There was at least a moderate correlation of septal and global mSASHA T1 and T2 values (*n* = 21) with MOLLI T1 (*n* = 17) and T2p-bSSFP T2 (*n* = 18) values (*r* > 0.6, *p* ≤ 0.003). OHTR patients without HR showed higher septal (T1: 1256 ± 42 ms vs. 1201 ± 46 ms, *p* = 0.002; T2: 47 ± 4 ms vs. 43 ± 2 ms, *p* = 0.003) and global mSASHA (T1: 1250 ± 58 ms vs. 1201 ± 45 ms, *p* = 0.013; T2: 48 ± 5 ms vs. 43 ± 2 ms, *p* = 0.009) values compared to normal controls (*n* = 20). In the control group, native mSASHA T1 and T2 values were higher in females (*n* = 9) than in males. Free-breathing mSASHA T2 mapping may be useful in identifying histological changes associated with ACAR, overcoming the challenges of breath holding and with good diagnostic performance.

**Supplementary Information:**

The online version contains supplementary material available at 10.1007/s10554-025-03582-9.

## Introduction

Free-breathing techniques expand the accessibility of cardiovascular magnetic resonance (CMR) assessment to a wider range of patients, but assessment of free-breathing relaxometry has been limited thus far. The breath-hold modified look-locker inversion-recovery (MOLLI) T1 and T2-prepared balanced steady-state free precession (T2p-bSSFP) T2 mapping sequences are commonly used for parametric mapping acquisition but are unreliable in unwell patients unable to co-operate with breath-holding.

Despite advances in immunosuppressive therapy, acute cardiac allograft rejection (ACAR) continues to be a significant and potentially life-threatening complication of heart transplantation. Although there is no absolute gold standard for the diagnosis of rejection, endomyocardial biopsy (EMB) with the histological assessment of acute cellular (ACR) and antibody-mediated rejection (AMR) is a key component of the clinical standard for rejection assessment. The invasive nature of EMB, associated adverse event rate, potential for sampling errors and interobserver variability in rejection grading have led to the investigation of less invasive techniques for decades [1].

**Fig. 1:**
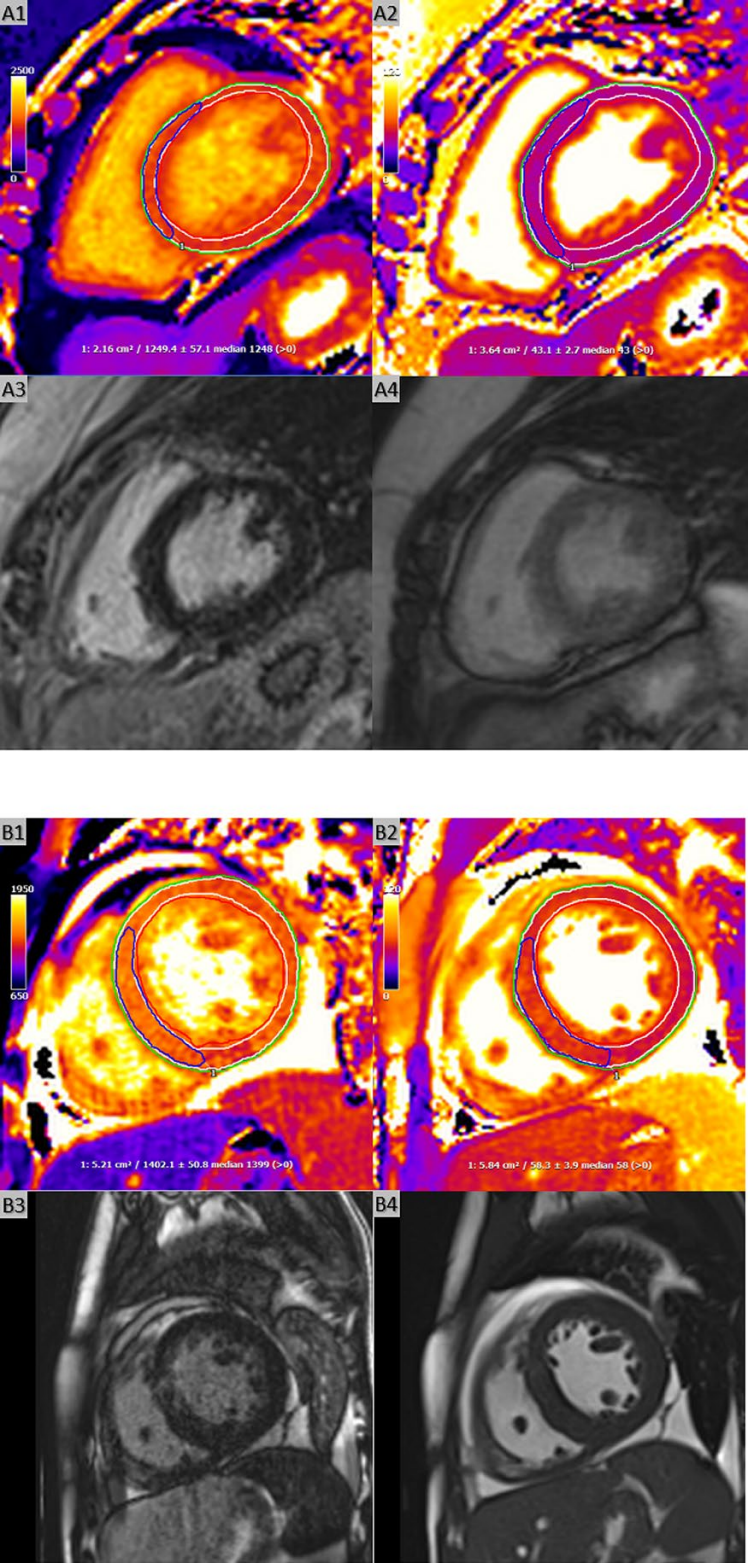
Mid-ventricular SAX slices of free-breathing mSASHA T1 (A1 and B1) and T2 (A2 and B2) maps, LGE images (A3 and B3), and cine images (A4 and B4). Endocardial (red lines) and epicardial (green lines) contours were drawn, with a 10% offset applied (white lines) to calculate global myocardial T1 and T2 values (A1–A2, B1–B2). ROIs were also placed in the septum (blue lines) to measure septal values (A1–A2, B1–B2). Pannel (A) represents a patient without HR. (A1) Septal and global mSASHA T1 values were 1249 and 1226 ms, respectively. (A2) Septal and global mSASHA T2 values were both 43 ms. (A3) No regions of LGE were observed. (A4) End-diastolic phase of cine images. Pannel (B) represents a patient with HR. (B1) Septal and global mSASHA T1 values were 1402 and 1371 ms, respectively. (B2) Septal and global mSASHA T2 values were 58 and 56 ms, respectively. (B3) LGE images demonstrated patchy enhancement in the mid-ventricular anteroseptum. (B4) End-diastolic phase of cine images, showing increased septal wall thickness (maximum 13 mm) and a pericardial effusion (~ 1 cm adjacent to the inferior wall in this slice). HR, histological rejection; LGE, late gadolinium enhancement; mSASHA, multiparametric saturation-recovery single-shot acquisition; ROI, region of interest; SAX, short axis

CMR is an important non-invasive method of providing accurate multiparametric information on this cohort of patients. Breath-holding T2p-bSSFP T2 and MOLLI T1 mapping acquisitions have long been used for investigating rejection due to correlations between T2 and T1 to myocardial oedema and fibrosis [2–4]. The novel free-breathing multiparametric saturation-recovery single-shot acquisition (mSASHA) T1 and T2 mapping sequence, may represent an alternative option when breath-holding ability is insufficient, as seen in acutely ill patients (Fig. 1). By enabling simultaneous acquisition of T1 and T2 mapping, mSASHA offers the unique advantages of co-registered parametric maps, reduced acquisition time, and fewer motion and registration artifacts. Compared to the MOLLI and T2p-bSSFP methods, the mSASHA technique generally yields higher T1 values and lower T2 values for normal myocardium [5].

The aim of the study was to compare free-breathing myocardial mSASHA [5] with established mapping techniques in heart transplant patients, evaluate its diagnostic performance in detecting histologically confirmed allograft rejection, and compare myocardial T1 and T2 values with those of healthy controls.

## Methods

This was a retrospective observational study of consecutive orthotopic heart transplant recipients (OHTRs) being investigated for potential allograft rejection who were referred for CMR scans. The study was approved by our institutional review board (IRAS project ID 292225). Patients with a CMR scan and an EMB within 1.5 month were identified and included in the study. EMBs were conducted as per an established protocol at our centre and were interpreted by experienced histopathologists according to 2005 International Society of Heart and Lung Transplantation (ISHLT) criteria [6]. AMR was graded based on the 2011 ISHLT consensus classification system [7]. The histological rejection (HR) group was defined as having any grade of ACR or AMR on EMB (ACR ≥ 1 R or AMR ≥ 1). Healthy participants served as the control group.

CMR scans were performed at 1.5T (MAGNETOM Aera, Siemens Healthineers, Forchheim, Germany). A standard CMR protocol with anatomical scout imaging, trans-axial balanced steady-state free precession and fast spin echo images, long and short axis (SAX) cine images, parametric T1 and T2 mapping, and long and short axis early and late gadolinium enhancement (LGE) images, was applied. Prototype free-breathing mSASHA mapping images and breath-holding T2p-bSSFP T2 and native MOLLI T1 mapping images were acquired in 4-chamber and mid SAX views (Supplementary Table 1). As described above, through concurrent T₁ and T₂ mapping acquisition, mSASHA shortens scanning time without the expense of motion and registration issues.

Post-contrast T1 mapping images (supplementary Table 1) were acquired 15 min after gadolinium agent injection (0.1 mmol/kg, Gadovist, Bayer). The septal mapping values were measured with regions of interest (ROIs) drawn in the septum in the mid SAX and 4-chamber images. The global mapping values were analysed in mid SAX slices by drawing epicardial and endocardial contours first and afterwards, by applying an off-set erosion of 10% in order to minimize partial voluming. Extracellular volume (ECV) was calculated using the pre-contrast T1 values of the myocardium (preT1_myo_) and the blood (preT1_blood_) and the post-contrast T1 values of the myocardium (postT1_myo_) and the blood (postT1_blood_) from the equation:

$$\begin{aligned}&ECV=\left( {1 - Haematocrit} \right)\times[(1/postT{1_{myo}}-{\text{ }}1/preT{1_{myo}})\\&\quad\quad\quad/(1/postT{1_{blood}} - 1/preT{1_{blood}})]\end{aligned}$$

LGE extent was measured as the area of myocardium with signal intensity greater than 3 standard deviations (SDs) above the mean of a reference ROI. Free breathing T1 and T2 mapping images, as described above, were also acquired in consecutive healthy participants, a cohort that was retrospectively included. All images were analysed by 2 cardiologists experienced in CMR (level 3 certified by the European Association of Cardiovascular Imaging) with cvi42 software (Circle Cardiovascular Imaging Inc., Version 6.0.3, Calgary, Canada). Image quality was assessed and considered diagnostic in all patients in the study, with interobserver reliability reported below. Analysis measurements were reported by consensus.

IBM SPSS Statistics Version 23 for Windows was used for the statistical analysis. Normal distribution of continuous variables was assessed graphically and with the Shapiro-Wilk normality test. Normally distributed variables are presented as mean ± SD and non-normally distributed variables as median [interquartile range (IQR)]. Inter-observer reliability was assessed using the intraclass correlation coefficient based on a two-way random-effects model with absolute agreement and single measurements [ICC(2,1)]. Student’s t-test for independent samples was used to assess differences between two normally distributed variables, whereas Mann-Whitney U test was used for two non-normal distributions. Correlations between two continuous variables were assessed with Pearson’s correlation coefficient. Pearson’s chi-square was used to assess differences between two dichotomous nominal variables, while Fisher’s exact test was used when the expected frequency in at least one cell of the corresponding 2 × 2 table was less than 5. Receiver operator characteristics (ROC) analysis and Youden’s index were used to assess the diagnostic performance of parametric mapping in identifying the rejection group. The level of statistical significance was set at *p* < 0.05.

## Results

Overall, 21 consecutive OHTRs (9 females, 43%) of a mean age of 43.2 ± 16.2 years were included in the study (Tables 1 and 2). The median time (IQR) since heart transplantation was 5.4 (1.2–14.8) years and the corresponding time interval between CMR and EMB was 5 (1–17) days. The most common cause of heart transplantation was dilated cardiomyopathy (12 patients, 57%). 12 patients (57%) had cardiac allograft vasculopathy (CAV), 13 (62%) had a past medical history of ACAR and 12 (57%) patients were IgG positive for cytomegalovirus (CMV). Treatment details are presented in Table 1. HR was identified in 9 patients (43%), of whom only 1 (11%) was female (*p* = 0.024).

**Table 1:** Characteristics of OHTRs and controls

	All patients (*n* = 21)	Non-HR group (*n* = 12)	HR group (*n* = 9)	*p* value*	Controls (*n* = 20)	*p* value**
Age (years)	43.2 ± 16.2	44.3 ± 13.5	41.8 ± 20.1	0.732	42.5 ± 12.6	0.880
Females [n (%)]	9 (43)	8 (67)	1 (11)	0.024	9 (45)	1.000
BMI (kg/m^2^)	27.4 ± 5.9	28.8 ± 5.8	25.6 ± 5.8	0.222	25.0 ± 5.9	0.189
BSA by Mosteller formula (m^2^)	1.98 ± 0.23	1.96 ± 0.24	2.00 ± 0.24	0.616	1.94 ± 0.26	0.591
Time of CMR since transplantation (years)	5.4 (1.2–14.8)	5.5 (0.9–15.2)	5.4 (1.4–14.8)	0.943		
Time interval between CMR and EMB (days)	5.0 (1.0–17.0)	5.5 (2.0–28.5)	5.0 (1.0–17.0)	0.544		
**Transplant indication**						
Dilated cardiomyopathy [n (%)]	12 (57)	7 (58)	5 (56)	0.630		
Ischaemic cardiomyopathy [n (%)]	3 (14)	1 (8)	2 (22)			
Other [n (%)]	6 (29)	4 (33)	2 (22)			
CAV [n (%)]	12 (57)	7 (58)	5 (56)	1.000		
History of rejection [n (%)]	13 (62)	6 (50)	7 (78)	0.367		
IgG CMV positive [n (%)]	12 (57)	7 (58)	5 (56)	1.000		
**Endomyocardial biopsy**						
ACR 0 and AMR 0 [n (%)]	12 (57)	12 (100)	0 (0)			
ACR 0 and AMR 1 [n (%)]	1 (5)	0 (0)	1 (11.1)			
ACR 1R and AMR 0 [n (%)]	4 (19)	0 (0)	4 (44)			
ACR 1R andAMR 1 [n (%)]	2 (10)	0 (0)	2 (22)			
ACR 2R and AMR0 [n (%)]	2 (10)	0 (0)	2 (22)			
**Immunosuppressant medications**						
Tacrolimus [n (%)]	13 (62)	8 (67)	5 (56)	0.673		
Sirolimus [n (%)]	4 (19)	2 (17)	2 (22)	1.000		
Everolimus [n (%)]	1 (5)	1 (8)	0 (0)	1.000		
Cyclosporine [n (%)]	3 (14)	1 (8)	2 (22)	0.553		
Mycophenolate mofetil [n (%)]	17 (81)	9 (75)	8 (89)	0.603		

* Non-HR vs. HR groups

**All patients vs. controls

ACR, acute cellular rejection; AMR, antibody-mediated rejection; BMI, body mass index; BSA, body surface area; CAV, cardiac allograft vasculopathy; CMV, cytomegalovirus; HR, histological rejection

**Table 2:** CMR findings in OHTRs and controls

	All patients (*n* = 21)	Non-HR group (*n* = 12)	HR group (*n* = 9)	*p* value*	Controls (*n* = 20)	*p* value**
**CMR findings**						
LVEDVI (ml/m^2^)	69.8 ± 15.3	66.5 ± 13.1	74.3 ± 17.6	0.256	69.8 ± 11.4	0.995
LVESVI (ml/m^2^)	24.7 (18.3–38.1)	23.9 (18.7–34.9)	25.9 (16.7–45.9)	0.887	24.1 (19.4–27.6)	0.434
LVEF (%)	60.0 (46.0–71.0)	59.5 (57.3–68.3)	68.0 (39.0–74.5)	0.695	65.5 (64.0–70.8)	0.117
LVMI (g/m^2^)	66.6 ± 16.3	60.3 ± 10.6	75.1 ± 19.1	0.035	53.0 ± 10.4	0.003
RVEDVI (ml/m^2^)	75.6 ± 19.3	74.6 ± 19.5	77.0 ± 20.2	0.781	78.1 ± 16.7	0.656
RVESVI (ml/m^2^)	34.6 ± 12.3	35.5 ± 12.8	33.4 ± 12.4	0.716	31.7 ± 8.2	0.381
RVEF (%)	54.2 ± 10.1	52.8 ± 8.0	56.0 ± 12.6	0.491	59.8 ± 3.3	0.025
**LGE**						
LGE mass (g)	0.24 (0–2.08)	0 (0–0.79)	1.19 (0.13–11.29)	0.054		
LGE (%)	0.30 (0–2.60)	0 (0–1.12)	0.90 (0.15–8.90)	0.088		
Presence of LGE [n (%)]	12 (57)	5 (42)	7 (78)	0.184		
Mid-wall LGE pattern (including septal insertion points) [n (%)]	8 (38)	3 (25)	5 (56)	0.203		
Subendocardial LGE pattern [n (%)]	4 (19)	3 (25)	1 (11)	0.603		
Transmural LGE pattern [n (%)]	2 (10)	0 (0)	2 (22)	0.171		
Septal insertion points LGE [n (%)]	6 (29)	3 (25)	3 (33)	1.000		
Septal LGE [n (%)]	6 (29)	2 (17)	4 (44)	0.331		
LV free wall LGE [n (%)]	6 (29)	2 (17)	4 (44)	0.331		

*Non-HR vs. HR groups

**Controls vs. non-HR groups

CMR, cardiovascular magnetic resonance; HR, histological rejection; LGE, late gadolinium enhancement; LV, left ventricular; LVEDVI, left ventricular end-diastolic volume index; LVEF, left ventricular ejection fraction; LVESVI, left ventricular end-systolic volume index; LVMI, left ventricular mass index; RVEDVI, right ventricular end-diastolic volume index; RVEF, right ventricular ejection fraction; RVESVI, right ventricular end-systolic volume index

**Fig. 2:**
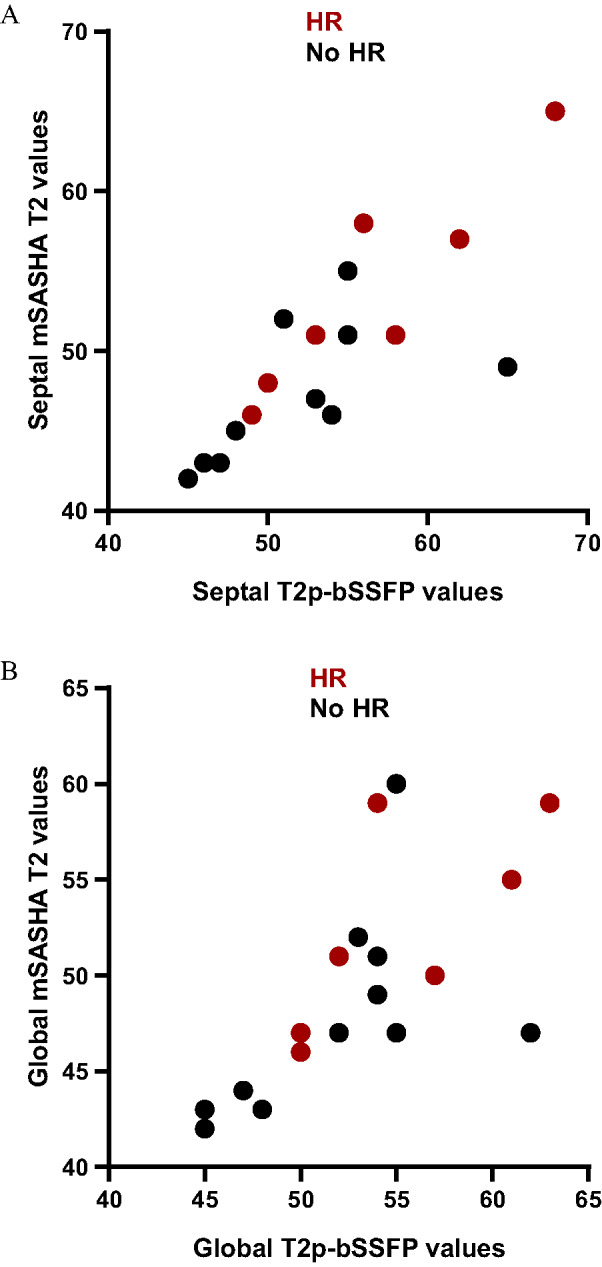
Scatter plots of septal (**A**) and global (**B**) mSASHA and T2p-bSSFP T2 mapping in OHRs. mSASHA values were correlated with T2p-bSSFP values (septal: *r* = 0.797, *p* < 0.001, *n* = 18; global: *r* = 0.670, *p* = 0.002, *n* = 18). HR, histological rejection; mSASHA, multiparametric saturation-recovery single-shot acquisition; T2p-bSSFP, T2-prepared balanced steady-state free precession

**Fig. 3:**
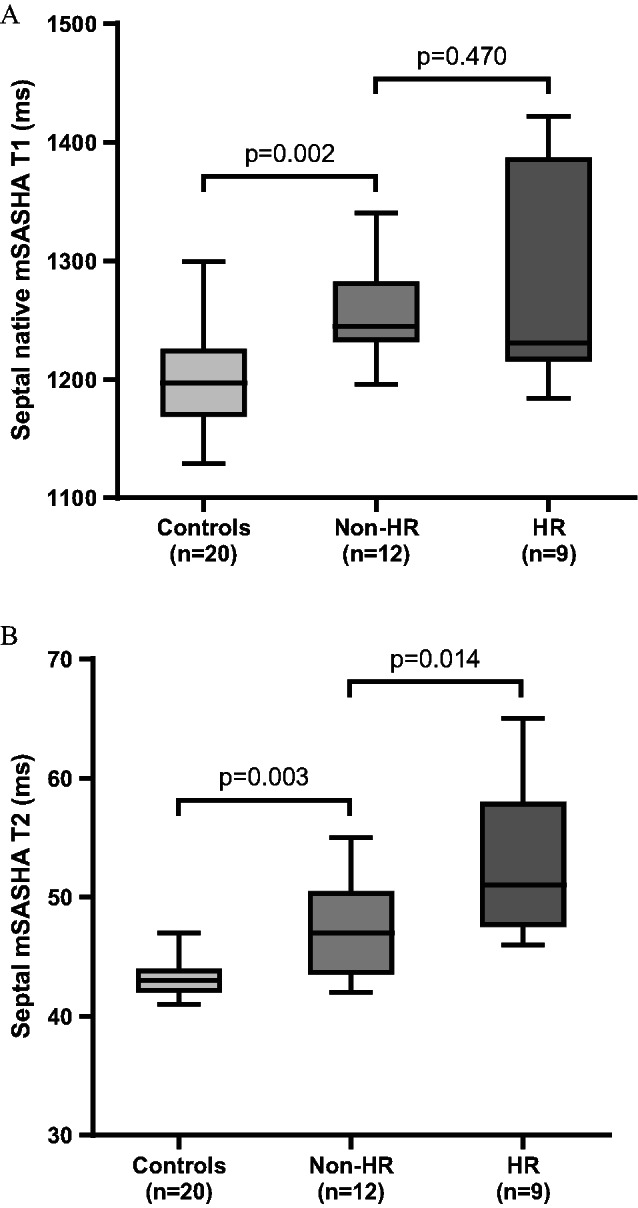
Free-breathing septal mSASHA native T1 and T2 values in controls (*n* = 20) and in non-HR (*n* = 12) and HR (*n* = 9) groups. HR, histological rejection; mSASHA, multiparametric saturation-recovery single-shot acquisition (A)

**Fig. 4:**
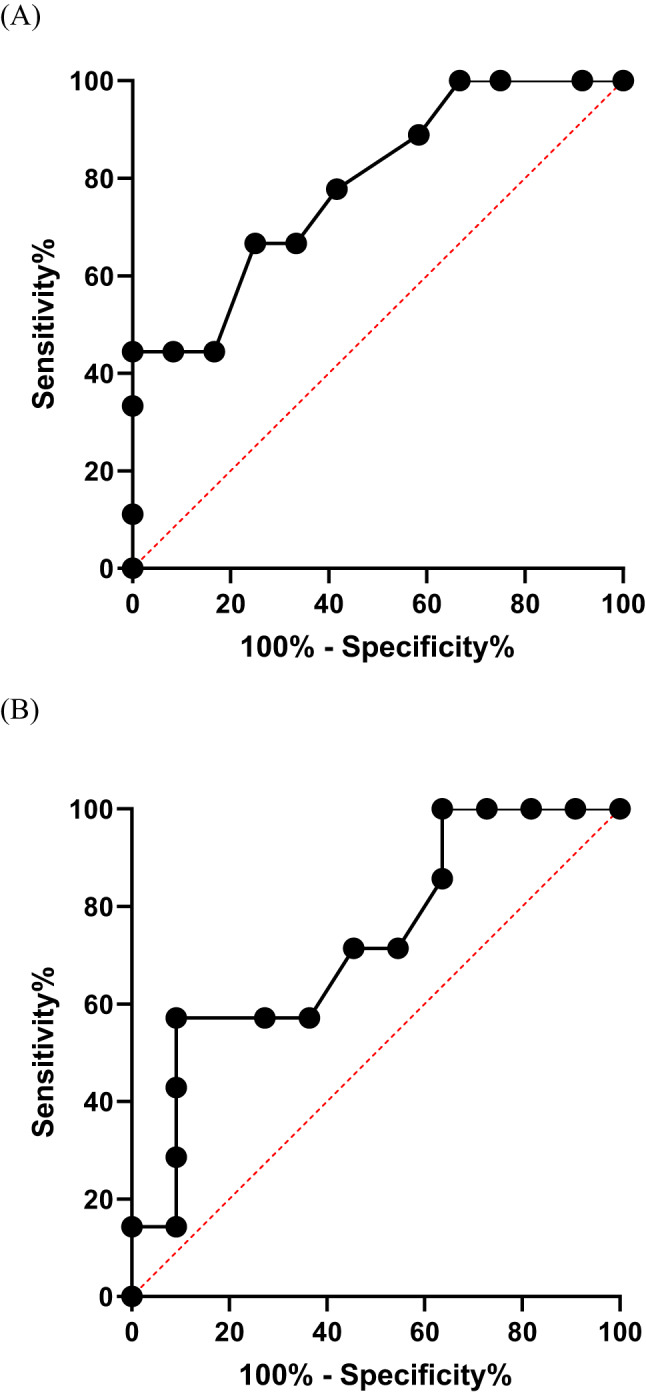
(**A**) ROC curve of free-breathing septal mSASHA T2 values in OHTRs (*n* = 21) in predicting patients with HR (*n* = 9): AUC = 0.79 (95%CI 0.59–0.98, *p* = 0.028). Youden’s index showed an optimal cut-off value of 50 ms (sensitivity 67%, specificity 75%). (**B**) Corresponding ROC analysis of breath-holding T2p-bSSFP T2 values (*n* = 18) failed to show statistically significant result in identifying patients with HR (*n* = 7): AUC = 0.73 (95%CI 0.52–0.93, *p* = 0.113)

Free-breathing myocardial mSASHA T1 and T2 mapping was acquired in all 21 OHTRs. Conventional breath-holding MOLLI T1 and T2p-bSSFP T2 mapping acquisitions were available in 17 and 18 patients, respectively. Measurements of mSASHA T₁ and T₂ values demonstrated good to excellent interobserver agreement [ICC(2,1) > 0.8, *p* < 0.001, supplementary Table 2]. Septal measurements showed excellent and higher interobserver reliability compared with global values. Septal and global mSASHA T1 and T2 values showed a moderate correlation with MOLLI T1 and T2p-bSSFP T2 values with a correlation coefficient r higher than 0.6 (Table 3; Fig. 2). The correlation was stronger for the septal T1 and T2 values compared to the global values, whereas the reverse was true for ECV. Septal mSASHA T2 values (Table 4; Fig. 3) were significantly higher in the HR (*n* = 9) group compared to the non-HR (*n* = 12) group (53 ± 6 ms vs. 47 ± 4 ms, *p* = 0.014). No significant difference was found in global mSASHA T2 values and in septal or global native mSASHA T1 or ECV values (Table 4; Fig. 3). The two groups did not show any significant difference in LGE extent (Table 2). In ROC analysis (Fig. 4), free-breathing septal mSASHA T2 values showed an area under the curve (AUC) of 0.79 [95% confidence interval (95%CI) 0.59–0.98, *p* = 0.028]. A cut-off value of 50 ms for septal mSASHA T2 values showed a sensitivity of 67% and specificity of 75% (Youden index J = 0.42) for identifying patients with HR. Breath-hold septal T2p-bSSFP T2 values (AUC = 0.73, 95%CI 0.52–0.93, *p* = 0.113) did not show any statistically significant difference in ROC analysis (18 OHTRs, 7 HR patients) (Fig. 4).

**Table 3:** Correlations (Pearson’s correlation coefficient r) between free-breathing mSASHA and breath-holding T2p-bSSFP, MOLLI, and T1-derived ECV mapping in the cohort of OHTRs

	Septal native MOLLI T1 (*n* = 17)	Global native MOLLI T1 (*n* = 17)	Septal T2p-bSSFP T2 (*n* = 18)	Global T2p-bSSFP T2 (*n* = 18)	Septal MOLLI ECV (*n* = 17)	Global MOLLI ECV (*n* = 17)	*p* value
Septal native mSASHA T1 (*n* = 17)	0.751						0.001
Global native mSASHA T1 (*n* = 17)		0.675					0.003
Septal mSASHA T2 (*n* = 18)			0.797				< 0.001
Global mSASHA T2 (*n* = 18)				0.670			0.002
Septal mSASHA ECV (*n* = 17)					0.751		0.001
Global mSASHA ECV (*n* = 17)						0.879	< 0.001

ECV, extracellular volume; MOLLI, modified look-locker inversion-recovery; mSASHA, multiparametric saturation-recovery single-shot acquisition; T2p-bSSFP, T2-prepared balanced steady-state free precession

**Table 4:** Free-breathing mSASHA mapping values and T1-derived ECV in OHTRs and in controls

	All patients (*n* = 21)	Non-HR group (*n* = 12)	HR group (*n* = 9)	*p* value*	Controls (*n* = 20)	*p* value**
Septal native mSASHA T1 values (ms)	1267 ± 67	1256 ± 42	1281 ± 92	0.470	1201 ± 46	0.002
Global native mSASHA T1 values (ms)	1251 ± 64	1250 ± 58	1252 ± 75	0.953	1201 ± 45	0.013
Septal mSASHA T2 values (ms)	50 ± 6	47 ± 4	53 ± 6	0.014	43 ± 2	0.003
Global mSASHA T2 values (ms)	50 ± 5	48 ± 5	52 ± 5	0.077	43 ± 2	0.009
Septal mSASHA ECV (%)	29 ± 5	28 ± 4	31 ± 6	0.180		
Global mSASHA ECV (%)	28 ± 4	27 ± 3	29 ± 4	0.247		

*Non-HR vs. HR groups

**Controls vs. non-HR groups

ECV, extracellular volume; HR, histological rejection; mSASHA, multiparametric saturation-recovery single-shot acquisition

The control group included 20 participants with native mSASHA T1 and T2 parametric mapping (MOLLI T1 and T2p-bSSFP T2 mapping acquisitions were available in 13 participants; T1-derived ECV was not available due to lack of HCT blood test measurement). Patients with no HR (*n* = 12) had significantly higher mSASHA T2 values (septal values 47 ± 4 vs. 43 ± 2, *p* = 0.003; global values 48 ± 5 vs. 43 ± 2, *p* = 0.009) compared with the controls (*n* = 20), as well as significantly higher native mSASHA T1 values [septal values 1256 ± 42 vs. 1201 ± 46, *p* = 0.002; global values 1250 ± 58 vs. 1201 ± 45, *p* = 0.013] (Table 4; Fig. 3). Similarly, the non-HR group had significantly higher breath-holding T2p-bSSFP T2 (*n* = 11) and native MOLLI T1 (*n* = 9) values compared to the controls (*n* = 13) (Table 5). In the control group, females (*n* = 9) had higher native mSASHA T1 and T2 values than males (*n* = 11) (Table 6); there was no significant correlation of mSASHA T1 and T2 values with age.

**Table 5:** Breath-holding T2p-bSSFP and MOLLI mapping values and T1-derived ECV in patients and in controls

	All patients (*n* = 18)	Non-HR group (*n* = 11)	HR group (*n* = 7)	*p* value*	Controls (*n* = 13)	*p* value**
Septal T2p-bSSFP T2 values (ms)	54 ± 6	52 ± 6	57 ± 7	0.121	47 ± 2	0.021
Global T2p-bSSFP T2 values (ms)	53 ± 5	52 ± 5	55 ± 5	0.185	47 ± 2	0.021
	All patient (*n* = 17)	Non-HR group (*n* = 9)	HR group (*n* = 8)		Controls (*n* = 13)	
Septal native MOLLI T1 values (ms)	1095 ± 75	1065 ± 44	1128 ± 91	0.085	1000 ± 28	< 0.001
Global native MOLLI T1 values (ms)	1086 ± 65	1066 ± 45	1107 ± 79	0.202	990 ± 22	< 0.001
Septal MOLLI ECV values (%)	33 ± 5	31 ± 3	35 ± 6	0.182		
Global MOLLI ECV values (%)	33 (30–34)	31 (30–33)	34 (29–37)	0.211		

* Non-HR vs. HR groups

** Controls vs. non-HR groups

ECV, extracellular volume; HR, histological rejection; MOLLI, modified look-locker inversion-recovery; T2p-bSSFP, T2-prepared balanced steady-state free precession

**Table 6:** CMR characteristics and free-breathing native mSASHA T1 and T2 values in controls

	All controls (*n* = 20)	Females (*n* = 9)	Males (*n* = 11)	*p* value
Age (years)	42.5 ± 12.6	45.9 ± 11.5	39.8 ± 13.3	0.289
Septal native mSASHA T1 (ms)	1201 ± 46	1231 ± 44	1177 ± 32	0.008
Global native mSASHA T1 (ms)	1201 ± 45	1238 ± 37	1171 ± 25	< 0.001
Septal mSASHA T2 (ms)	43 ± 2	44 ± 2	42 ± 1	0.025
Global mSASHA T2 (ms)	43 ± 2	45 ± 2	42 ± 1	0.001
LVEDVI (ml/m^2^)	69.8 ± 11.4	63.3 ± 5.9	75.1 ± 12.2	0.017
LVESVI (ml/m^2^)	23.4 ± 4.9	20.7 ± 4.4	25.7 ± 4.2	0.018
LVEF (%)	66.5 ± 4.3	67.7 ± 4.9	65.5 ± 3.7	0.284
LVMI (g/m^2^)	53.0 ± 10.4	44.8 ± 4.7	59.8 ± 8.9	< 0.001
RVEDVI (ml/m^2^)	78.1 ± 16.7	68.2 ± 9.4	86.3 ± 17.2	0.011
RVESVI (ml/m^2^)	31.7 ± 8.2	25.9 ± 4.3	36.4 ± 7.6	0.002
RVEF (%)	59.8 ± 3.3	62.1 ± 2.5	57.8 ± 2.5	0.001

CMR, cardiovascular magnetic resonance; LVEDVI, left ventricular end-diastolic volume index; LVEF, left ventricular ejection fraction; LVESVI, left ventricular end-systolic volume index; LVMI, left ventricular mass index; mSASHA, multiparametric saturation-recovery single-shot acquisition; RVEDVI, right ventricular end-diastolic volume index; RVEF, right ventricular ejection fraction; RVESVI, right ventricular end-systolic volume index

## Discussion

CMR is a powerful instrument for the evaluation of the heart, and free-breathing techniques have been deployed and tested for use in a broad patient population. Breath-hold MOLLI T1 and T2p-bSSFP T2 mapping sequences are commonly used for parametric mapping acquisition. A free-breathing approach to the assessment of parametric mapping would extend the ability to evaluate T1 and T2 relaxation times in unwell patients unable to co-operate with breath-holding. These patients are often amongst those who are most likely to benefit from the identification of potentially reversible cardiac inflammation. We demonstrate that free-breathing mSASHA T2 is better able to detect EMB defined rejection than conventional T2p-bSSFP without trade-offs in image quality.

EMB has been a cornerstone of the clinical approach to rejection assessment since the early 1970 s [8]. Although mild rejection is usually not clinically or prognostically significant, the use of histology provides a unique biological reference for comparison to CMR parametric myocardial indices. CMR, which allows for myocardial tissue characterization and evaluation of cardiac structure and function, has been studied as a non-invasive alternative to EMB with variable results in this diffuse disease. Myocardial T1 and T2 are both influenced by water content and thus tend to be both elevated in case of oedema, which may occur as a consequence of inflammatory response to ACAR. Moreover, the interplay between T1 and T2 depends on the molecular content such as protein quantity and extracellular matrix extent. It is noteworthy that, although EMB serves as the histological reference standard, sampling errors may lead to underestimation of parametric CMR value elevations.

Older T2-weighted spin-echo sequences were not able to show consistent results in detecting ACAR [8]. Later studies using respiratory-navigated turbo spin echo and breath-holding T2p-bSSFP T2 mapping acquisitions revealed the value of T2 mapping in ACAR, particularly when combined with T1 mapping or T1 mapping-derived ECV [1, 2, 4]. Inconsistent results have also been published for T1 mapping, typically performed using the breath-holding MOLLI sequence [1, 2, 4]. Reasons for variability in results included differing study designs, alongside definitions of ACAR, and variations in manufacturer, sequences and methods used for T1 and T2 mapping measurements (global, segmental, peak segmental or septal values). Such inconsistencies across published data may thus contribute to the lack of inclusion in CMR within current ISHLT guidelines for the assessment of ACAR. To provide a “proof of concept” assessment for a novel free-breathing sequence, our study focused on comparison with histological rejection as the gold standard for assessing the performance of mSASHA in detecting tissue oedema, rather than clinical rejection or in predicting outcomes, to minimise the heterogeneity of clinical judgement in a disease characterised by recurrent subacute episodes on top of chronic presentations.

mSASHA is a simultaneous T1 and T2 mapping technique, hence multiparametric with both T1 and T2 acquisition in one scan (Supplementary Table 1). This is achieved by combining saturation-recovery and T2-preparation modules, typically acquiring one non-prepared reference, several saturation-recovery images, and then saturation-plus-T2-prepared images. MOLLI 5(3)3 is a T1 mapping technique that acquires images along T1-recovery after inversion recovery pulses, using a scheme of 5 images after first inversion pulse, a 3-beat pause, then 3 images after second inversion pulse, all within one breath-hold. Three-point T2 mapping technique acquires images with different T2-preparation times (0, 25, and 55 ms), fitting the signal decay across these points to estimate T2 in a single breath-hold. Simultaneous T1 and T2 mapping with mSASHA shortens scan time: native mSASHA acquires both maps in 11 heartbeats, versus 11 heartbeats for a MOLLI 5(3)3 native T1 map plus 11 heartbeats for a T2p-bSSFP T2 map (total 22 heartbeats).

Previous breath-hold mSASHA use has shown high accuracy in myocardial T1 and T2 quantification in volunteers at 3 T [5]. When tested on paediatric OHTRs, free-breathing mSASHA T1 and T2 mapping could identify myocardial regions with elevated T1 and T2 values found on conventional corresponding MOLLI and T2p-bSSFP maps with high sensitivity and specificity [9].

To the best of our knowledge, this is the first study investigating the performance of the novel free-breathing mSASHA technique in adult OHTRs and in diffuse histological ACAR. Septal and global mSASHA T2 mapping values showed a moderate correlation (*r* > 0.60) with the conventional T2p-bSSFP T2 values. Native mSASHA T1 mapping and T1-derived ECV also showed at least a moderate correlation (*r* > 0.60) with MOLLI-derived measurements. The limited ability of some patients to comply with breath-holding instructions may have contributed to weaker correlations than expected (true T1 or T2 values cannot be directly measured). Septal T1 and T2 values demonstrated stronger correlations than global values, while global ECV values showed stronger correlation than septal ECV values. Global ECV values derived with mSASHA and MOLLI showed the strongest correlation (*r* = 0.879, *p* > 0.001). This may be attributed to the fact that global ECV is derived by averaging values across multiple myocardial segments, reducing the impact of regional artifacts and noise, and relies on relative changes between pre- and post-contrast T1 values, making it less sensitive to absolute T1 variability in a single region.

Free-breathing septal mSASHA T2 was the only mapping parameter which was significantly higher in patients with any HR (ACR ≥ 1 R or AMR ≥ 1) compared to those with a negative ACR and AMR biopsy grading, with an optimal cut-off T2 value identified at 50 ms. In our cohort of sick patients, mSASHA T2 mapping was superior to breath-holding T2p-bSSFP in differentiating patients with HR, although a similar trend of greater T2 values in HR was present without statistical significance. The mSASHA T2 estimates of myocardial oedema provided in this study are unique, as the mapping sequence independently and accurately estimates T1 and T2 simultaneously as opposed to sequentially, without T1 confounding T2 or T1 confounding T2 [10]. The signal for each image is always reset to zero longitudinal magnetization with a saturation pulse, reducing carryover confounders. mSASHA T2 measurements are therefore independent of T1 and heart rate, unlike T2p-bSSFP [9]; independence from heart rate is particularly important in OHTRs who may exhibit baseline tachycardia due to parasympathetic denervation and upregulation of β2-adrenergic receptors in the transplanted heart [11].

Also, T1 and T2 values are derived from separate signal intensities controlled by different timing schemes (saturation recovery vs. T2-preparation) so there is no potential bias caused by one relaxation time in the fitting of the other. The use of motion-corrected single-shot readouts during steady free-breathing with mSASHA improves the patient experience compared to traditional breath-holds, while also increasing precision due to the increased number of images acquired. The increased accuracy and precision of mSASHA T2 may therefore explain the increased diagnostic performance compared to conventional T2p-bSSFP.

Global mSASHA T2 values also trended toward increased values in patients with HR but did not reach statistical significance like with septal mSASHA T2 values. Septal values may have been more robustly assessed than global, given the lower likelihood of partial volume errors from ROIs drawn within the thicker septum, compared to measurements derived from the thinner lateral wall encompassed within global mSASHA measurements [12]. The inclusion of a control group in our study demonstrates that patients with no HR had higher native T1 and T2 values than controls, possibly reflecting established fibrosis related to previous rejection or/and low-grade chronic myocardial inflammation. The control group also provides reference mSASHA T1 and T2 mapping values, with females showing higher values than males, consistent with findings from conventional T1 and T2 sequences [13].

A recent meta-analysis reported pooled sensitivities of 86%, 84%, and 90%, and pooled specificities of 79%, 82%, and 67% for detecting ACAR (including 143 events with ≥ 1R ACAR) [14]. However, heterogeneity in methodologies, such as variations in the acquisition and measurement of native T1, T2, and ECV mapping, as well as differences in ACAR definitions, may account for the discrepancies between these results and our study.

## Limitations

The limitations of the present study include the retrospective collection of data and the small sample size. Additionally, the results should be interpreted in the context of the time interval between CMR and EMB, and of the immunosuppressant treatment being received. The cardiac phase of acquisition might also affect relaxometry values, including those from our reference cohort. Regional parametric mapping values were acquired exclusively from the septum, which, along with global values, has demonstrated higher reproducibility for both T1 and T2 mapping compared to the inferior, lateral, and anterior walls [12]. The lateral wall is more susceptible to motion artifacts, partial volume effects, and B0/B1 field inhomogeneities, whereas the mid-ventricular septum, due to its central and stable position, is less affected by these limitations and is generally preferred [15]. The need for cautious extrapolation of the above findings thus underscores the requirement for larger studies to improve practical applicability.

## Conclusion

Free-breathing mSASHA T2 mapping shows a moderate correlation with the conventional breath-hold T2p-bSSFP T2 mapping in heart transplant recipients and may be useful in identifying patients with histological rejection, improving accessibility of non-invasive oedema assessment to this patient cohort.

## Supplementary Information

Below is the link to the electronic supplementary material.

Supplementary Material 1

Supplementary Material 2

## Data Availability

No datasets were generated or analysed during the current study.

## Abbreviations

95%CI: 95% confidence interval
ACAR: Acute cardiac allograft rejection
ACR: Acute cellular rejection
AMR: Antibody-mediated rejection
AUC: Area under the curve
BMI: Body mass index
BSA: Body surface area
CAV: Cardiac allograft vasculopathy
CMR: Cardiovascular magnetic resonance
CMV: Cytomegalovirus
ECV: Extracellular volume
EMB: Endomyocardial biopsy
HR: Histological rejection
ICC(2,1): Intraclass correlation coefficient based on a two-way random-effects model with absolute agreement and single measurements
IQR: Interquartile range
LGE: Late gadolinium enhancement
LV: Left ventricular
LVEDVI: Left ventricular end-diastolic volume index
LVEF: Left ventricular ejection fraction
LVESVI: Left ventricular end-systolic volume index
LVMI: Left ventricular mass index
MOLLI: Modified look-locker inversion-recovery
mSASHA: Multiparametric saturation-recovery single-shot acquisition
OHTR: Orthotopic heart transplant recipient
postT1_blood_: Post-contrast T1 values of the blood
postT1_myo_: Post-contrast T1 values of the myocardium
preT1_blood_: Pre-contrast T1 values of the blood
preT1_myo_: Pre-contrast T1 values of the myocardium
ROC: Receiver operator characteristics
ROI: Region of interest
RVEDVI: Right ventricular end-diastolic volume index
RVEF: Right ventricular ejection fraction
RVESVI: Right ventricular end-systolic volume index
SAX: Short axis
SD: Standard deviation
T2p-bSSFP: T2-prepared balanced steady-state free precession

## References

[1] Anthony C, Imran M, Pouliopoulos J, Emmanuel S, Iliff J, Liu Z et al (2022) Cardiovascular magnetic resonance for rejection surveillance after cardiac transplantation. Circulation 145(25):1811–1824. 10.1161/CIRCULATIONAHA.121.057006 35621277 10.1161/CIRCULATIONAHA.121.057006

[2] Dolan RS, Rahsepar AA, Blaisdell J, Suwa K, Ghafourian K, Wilcox JE et al (2019) Multiparametric cardiac magnetic resonance imaging can detect acute cardiac allograft rejection after heart transplantation. JACC Cardiovasc Imaging 12(8 Pt 2):1632–1641. 10.1016/j.jcmg.2019.01.026 30878427 10.1016/j.jcmg.2019.01.026 PMC6995349

[3] Imran M, Wang L, McCrohon J, Yu C, Holloway C, Otton J et al (2019) Native T1 mapping in the diagnosis of cardiac allograft rejection: A prospective histologically validated study. JACC Cardiovasc Imaging 12(8 Pt 2):1618–1628. 10.1016/j.jcmg.2018.10.027 30660547 10.1016/j.jcmg.2018.10.027

[4] Vermes E, Pantaléon C, Auvet A, Cazeneuve N, Machet MC, Delhommais A et al (2018) Cardiovascular magnetic resonance in heart transplant patients: diagnostic value of quantitative tissue markers: T2 mapping and extracellular volume fraction, for acute rejection diagnosis. J Cardiovasc Magn Reason 20(1):59. 10.1186/s12968-018-0480-9 10.1186/s12968-018-0480-9 PMC6114788 30153847

[5] Chow K, Hayes G, Flewitt JA, Feuchter P, Lydell C, Howarth A et al (2022) Improved accuracy and precision with three-parameter simultaneous myocardial T1 and T2 mapping using multiparametric SASHA. Magn Reson Med 87(6):2775–2791. 10.1002/mrm.29170 35133018 10.1002/mrm.29170

[6] Stewart S, Winters GL, Fishbein MC, Tazelaar HD, Kobashigawa J, Abrams J et al (2005) Revision of the 1990 working formulation for the standardization of nomenclature in the diagnosis of heart rejection. J Heart Lung Transpl 24(11):1710–1720. 10.1016/j.healun.2005.03.019 10.1016/j.healun.2005.03.019 16297770

[7] Kobashigawa J, Crespo-Leiro MG, Ensminger SM, Reichenspurner H, Angelini A, Berry G et al (2011) Report from a consensus conference on antibody-mediated rejection in heart transplantation. J Heart Lung Transpl 30(3):252–269. 10.1016/j.healun.2010.11.003 10.1016/j.healun.2010.11.003 PMC3829685 21300295

[8] Butler CR, Thompson R, Haykowsky M, Toma M, Paterson I (2009) Cardiovascular magnetic resonance in the diagnosis of acute heart transplant rejection: a review. J Cardiovasc Magn Reson 11(1):7. 10.1186/1532-429X-11-7 19284612 10.1186/1532-429X-11-7 PMC2660322

[9] Richmann DP, Contento J, Cleveland V, Hamman K, Downing T, Kanter J et al (2024) Accuracy of free-breathing multi-parametric SASHA in identifying T1 and T2 elevations in pediatric orthotopic heart transplant patients. Int J Cardiovasc Imaging 40(1):83–91. 10.1007/s10554-023-02965-0 37874446 10.1007/s10554-023-02965-0 PMC10842347

[10] Kellman P, Xue H, Chow K, Howard J, Chacko L, Cole G et al (2021) Bright-blood and dark-blood phase sensitive inversion recovery late gadolinium enhancement and T1 and T2 maps in a single free-breathing scan: an all-in-one approach. J Cardiovasc Magn Reson 23(1):126. 10.1186/s12968-021-00823-3 34743718 10.1186/s12968-021-00823-3 PMC8573877

[11] Farrukh HM, White M, Port JD, Handwerger D, Larrabee P, Klein J, Roden RA, Skerl L, Renlund DG, Feldman AM et al (1993) Up-regulation of beta 2-adrenergic receptors in previously transplanted, denervated nonfailing human hearts. J Am Coll Cardiol 22(7):1902–1908. 10.1016/0735-1097(93)90777-x 8245347 10.1016/0735-1097(93)90777-x

[12] Böttcher B, Lorbeer R, Stöcklein S, Beller E, Lang CI, Weber MA et al (2021) Global and regional Test-Retest reproducibility of native T1 and T2 mapping in cardiac magnetic resonance imaging. J Magn Reson Imaging 54(6):1763–1772. 10.1002/jmri.27755 34075646 10.1002/jmri.27755

[13] Granitz M, Motloch LJ, Granitz C, Meissnitzer M, Hitzl W, Hergan K et al (2019) Comparison of native myocardial T1 and T2 mapping at 1.5T and 3T in healthy volunteers: reference values and clinical implications. Wien Klin Wochenschr 131(7–8):143–155. 10.1007/s00508-018-1411-3 30519737 10.1007/s00508-018-1411-3 PMC6459801

[14] Anthony C, Wang TKM, Agrawal A, Alvarez P, Estep JD, Xu B (2025) Diagnostic performance of native T1, T2, and extracellular volume mapping for cardiac allograft rejection. JACC Heart Fail 13(9):102554. 10.1016/j.jchf.2025.102554 40644947 10.1016/j.jchf.2025.102554

[15] Messroghli DR, Moon JC, Ferreira VM, Grosse-Wortmann L, He T, Kellman P et al (2017) Clinical recommendations for cardiovascular magnetic resonance mapping of T1, T2, T2* and extracellular volume: A consensus statement by the society for cardiovascular magnetic resonance (SCMR) endorsed by the European association for cardiovascular imaging (EACVI). J Cardiovasc Magn Reson 19(1):75. 10.1186/s12968-017-0389-8 28992817 10.1186/s12968-017-0389-8 PMC5633041

