# Supplementary material for: The performance of free-breathing multiparametric SAturation-recovery single-SHot acquisition T1 and T2 mapping in cardiac allograft rejection
Source: Int J Cardiovasc Imaging. 2025 Dec 12;42(1):149–59. doi: 10.1007/s10554-025-03582-9 (PMC12847199; doi:10.1007/s10554-025-03582-9)
Supplement: Supplementary file 1 — Supplementary Material 1 [file 10554_2025_3582_MOESM1_ESM.docx]

| **Parameter** | **Pre-contrast**  **MOLLI T1** | **Pre-contrast**  **mSASHA** | **T2p-bSSFP T2** | **Post-contrast**  **MOLLI T1** | **Post-contrast**  **mSASHA** |
| --- | --- | --- | --- | --- | --- |
| Acquisition | Single shot SSFP | Single shot SSFP | Single shot SSFP | Single shot SSFP | Single shot SSFP |
| Sampling Scheme | 5 (3) 3  TI increment: 80 ms | TS: 550 ms | 0, 25, 55 ms T2p duration | 4 (1) 3 (1) 2  TI increment: 80 ms | TS: 300 ms |
| Flip angle (^o^) | 35 | 100  maximum variable flip angle | 70 | 35 | 100  maximum variable flip angle |
| Field of view (mm^2^) | 360 x 270 | 360 x 270 | 360 x 270 | 360 x 270 | 360 x 270 |
| Image matrix | 256 x 144 | 256 x 144 | 192 x 120 | 256 x 144 | 256 x 144 |
| Echo time (ms) | 1.12 | 1.26 | 1.06 | 1.12 | 1.26 |
| Bandwidth (Hz/pixel) | 1085 | 1085 | 1185 | 1085 | 1085 |
| Parallel acquisition/acceleration factor | GRAPPA/2 | GRAPPA/2 | GRAPPA/2 | GRAPPA/2 | GRAPPA/2 |
| Partial Fourier | 7/8 | 7/8 | 7/8 | 7/8 | 7/8 |
| Slice thickness (mm) | 8 | 8 | 8 | 8 | 8 |
| Number of segments | 72 | 72 | 60 | 72 | 72 |
| Echo spacing (ms) | 2.7 | 2.9 | 2.5 | 2.7 | 2.9 |
| Acquisition time | 11 heart beats | 11 heart beats | 11 heart beats | 11 heart beats | 11 heart beats |

Supplementary Table 1

Parameters of parametric mapping acquisitions.

GRAPPA, generalized autocalibrating partially parallel acquisition

MOLLI, modified look-locker inversion-recovery

mSASHA, multiparametric saturation-recovery single-shot acquisition

SSFP, steady-state free precession

T2p, T2 prepared

T2p-bSSFP, T2-prepared balanced steady-state free precession

TI, inversion time

TS, saturation recovery time
