# Supplementary material for: The performance of free-breathing multiparametric SAturation-recovery single-SHot acquisition T1 and T2 mapping in cardiac allograft rejection
Source: Int J Cardiovasc Imaging. 2025 Dec 12;42(1):149–59. doi: 10.1007/s10554-025-03582-9 (PMC12847199; doi:10.1007/s10554-025-03582-9)
Supplement: Supplementary file 2 — Supplementary Material 2 [file 10554_2025_3582_MOESM2_ESM.docx]

| **Parameter** | **ICC(2,1)** | **95%CI** | **p value** |
| --- | --- | --- | --- |
| Septal mSASHA T2 | 0.98 | 0.96-0.99 | <0.001 |
| Global mSASHA T2 | 0.91 | 0.80-0.96 | < 0.001 |
| Septal mSASHA T1 | 0.98 | 0.94-0.99 | < 0.001 |
| Global mSASHA T1 | 0.84 | 0.63-0.93 | < 0.001 |

Supplementary Table 2

Inter-observer reliability between the 2 cardiologists performing the CMR analysis. There was good to excellent interobserver agreement, with septal values demonstrating excellent and higher agreement compared with global values.

95%CI, 95% confidence interval; ICC(2,1), Intraclass correlation coefficient based on a two-way random-effects model with absolute agreement and single measurements; mSASHA, multiparametric saturation-recovery single-shot acquisition.
